# A neuromorphic multi-scale approach for real-time heart rate and state detection

**DOI:** 10.1038/s44335-025-00024-6

**Published:** 2025-04-02

**Authors:** Chiara De Luca, Mirco Tincani, Giacomo Indiveri, Elisa Donati

**Affiliations:** 1 Institute of Neuroinformatics, University of Zurich and ETH Zurich, Zurich, Switzerland; 2 Digital Society Initiative, University of Zurich, Zurich, Switzerland

**Keywords:** Electrical and electronic engineering, Information theory and computation, Computational science

## Abstract

With the advent of novel sensor and machine learning technologies, it is becoming possible to develop wearable systems that perform continuous recording and processing of biosignals for health or body state assessment. For example, modern smartwatches can already track physiological functions, including heart rate and its anomalies, with high precision. However, stringent constraints on size and energy consumption pose significant challenges for always-on operation to detect trends across multiple time scales for extended periods of time. To address these challenges, we propose an alternative solution that exploits the ultra-low power consumption features of mixed-signal neuromorphic technologies. We present a biosignal processing architecture that integrates multimodal sensory inputs and processes them using the principles of neural computation to reliably detect trends in heart rate and physiological states. We validate this architecture on a mixed-signal neuromorphic processor and demonstrate its robust operation despite the inherent variability of the analog circuits present in the system. In addition, we demonstrate how the system can process multi scale signals, namely instantaneous heart rate and its long-term states discretized into distinct zones, effectively detecting monotonic changes over extended periods that indicate pathological conditions such as agitation. This approach paves the way for a new generation of energy-efficient stand-alone wearable devices that are particularly suited for scenarios that require continuous health monitoring with minimal device maintenance.

## Introduction

Biological neural processing systems can process analog sensory signals and manipulate discrete elements over multiple time scales using a common computational substrate that is extremely energy efficient, but also highly heterogeneous and variable^1–3^. Neuromorphic analog circuits that emulate the physics of real neurons are also affected by device variability and noise sensitivity^4,5^; however, they have similar advantages in terms of power consumption and support for rich dynamics and state-dependent computation^6,7^.

By using populations of neurons, biological neural networks can achieve robust and reliable computation^8,9^: In the sensory processing domain, this strategy allows them to perform statistical inference by computing likelihood functions^10,11^, while at higher levels of processing, they can be configured to manipulate discrete symbols and states over longer time scales by forming attractors and working memory networks^1,12–14^.

Similar to biological systems, mixed-signal analog/digital neuromorphic processing systems can exploit the collective behavior of neuron populations distributed over multiple cores to achieve robust and reliable computation despite inherent variability and noise^7,15^. This approach has been successfully applied in various healthcare domains^16–18^, including biosignal processing, where neuromorphic architectures have demonstrated the ability to detect anomalies in Electrocardiography (ECG) recordings^19,20^, classify Electromyography (EMG) signals^21–23^, or detect relevant biomarkers in Electroencephalography (EEG) data measured from epileptic patients^24–26^.

In this paper, we demonstrate how populations of silicon neurons implemented using analog neuromorphic circuits can be configured and programmed to perform complex biosignal processing tasks involving multimodal sensory fusion, multi-timescale analysis, and state-dependent computation. This approach addresses a critical need for energy-efficient systems capable of continuously monitoring physiological signals in real time, a requirement that is particularly relevant for applications such as health monitoring in elderly people^27^ or individuals with cognitive impairments^28^.

Detecting Heart Rate (HR) changes, especially monotonic trends over prolonged periods, is critical to understanding cardiovascular health, tracking fitness levels, and identifying early signs of pathological conditions such as arrhythmias or stress-induced states^29,30^. Smartwatches, such as the Apple Watch, have demonstrated their ability to continuously monitor physiological signals and have even been used in clinical trials to detect pathologies such as atrial fibrillation^31^. Some advanced models are evolving into medical-grade devices and are likely to play a significant role in future healthcare^32^. However, despite these advances, smartwatches face significant limitations in terms of power efficiency and scalability. Processing multiple inputs, defining physiological states, and tracking long-term trends often requires significant computational resources that quickly drain the battery. In addition, many functionalities rely heavily on cloud-based infrastructure for long-term data storage and analysis, raising privacy concerns and limiting their utility in scenarios where constant connectivity is impractical^33^. These limitations make smartwatches and similar devices less suitable for long-term, continuous monitoring in low-power scenarios or for individuals who cannot reliably manage device maintenance, such as elderly patients or those with dementia.

Designing solutions that address both power efficiency and prolonged usability in wearable heart rate sensors is an active area of research. Various strategies, ranging from algorithmic refinements to specialized hardware and sensor placement, are being investigated^18,34,35^. On the deep learning side, combining Temporal Convolutional Networks with hardware-friendly quantization provides highly accurate HR tracking while minimizing energy consumption^36^. Alternatively, an adaptive heartbeat locked loop that dynamically adjusts system parameters based on HR variations achieves up to a 3.3-fold reduction in power consumption compared to traditional approaches,an essential advantage for battery-sensitive scenarios^37^.

Our proposed solution builds on this foundation by leveraging neuromorphic technology to perform signal filtering, subtraction, and heart rate computation directly on analog hardware, removing the need for extensive software overhead or cloud-based infrastructure. Inspired by canonical cortical microcircuits^38,39^, we developed a neural architecture that integrates sensory signals from multiple sources, such as accelerometers, Photoplethysmography (PPG), and ECG and processes them using biologically plausible spike-based computational primitives, including soft Winner-Take-All (sWTA) networks^40,41^ and Neural State Machines (NSMs)^6,42^. This system employs a multi-timescale processing strategy: short-term processing for real-time HR decoding, mid-term processing for HR zone detection, and long-term processing to track slow monotonic changes in HR trends over extended periods. Importantly, this entire functionality is achieved on the same neuromorphic hardware substrate without altering its parameters or conditions. Our approach enables the device to operate seamlessly across these three time scales, which is a novel contribution not addressed by existing state-of-the-art methods. By leveraging this approach, the system not only maintains low power consumption and long-term usability but also reliably detects monotonic trends in HR over extended periods, ensuring robust and efficient performance.

We validated this approach using a prototype mixed-signal neuromorphic chip^15^, and obtained experimental results that demonstrate the system’s ability to perform the desired computation reliably with low-power. The hardware network achieves robust computation despite the variability in the analog circuits and noise in the input signals, thanks to the adopted strategy of exploiting neuron populations and their collective dynamics^7^. The results obtained highlight the potential of the proposed neuromorphic approach to enable the construction of ultra-low-power wearable systems specifically tailored for medical applications that cannot rely on cloud-based computing or devices that require frequent charging, such as smartwatches. This is particularly important for scenarios involving vulnerable populations such as infants, the elderly, or animal welfare, where consistent device operation is essential but practical constraints limit frequent maintenance or connectivity.

Another feature that sets this system apart is its ability to monitor multiple physiological signals continuously and go beyond conventional heart rate detection. By encoding multiple sensory inputs with address-events^43^ and integrating them into a common spiking neural network framework, the proposed neural processing architecture can infer broader physiological states, enabling applications ranging from activity detection in fitness tracking to early detection of stress or agitation in dementia care.

In summary, we propose a novel event-based processing architectures that is compatible with ultra-low-power neuromorphic hardware and adopts principles of neural design to achieve robust computation^3^. Unlike traditional systems that rely on software-driven post-processing^44,45^, and that consequently have substantial energy consumption and memory storage demands, the proposed architecture processes data locally and in real time, eliminating the need for high-resolution storage or energy-intensive computing resources. As a result, it significantly reduces power consumption while enabling longer duration monitoring without compromising accuracy. By addressing the limitations of conventional digital implementations, this work represents a transformative step forward in wearable technology, paving the way for practical, always-on monitoring in both clinical and non-clinical settings.

## Results

**Fig. 1:**
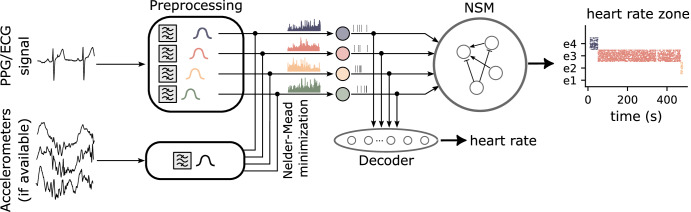
Overview of the neuromorphic spiking network architecture and signal processing pipeline. Input signals (Photoplethysmography (PPG), Electrocardiography (ECG), and accelerometer data, if available) are preprocessed through a series of bandpass filters to isolate distinct frequency components. When denoising is required, signals from accelerometers and PPG are linearly combined to reduce motion artifacts (with parameters optimized by the Nelder-Mead method^48^). Each filtered band is then amplified and sent to a separate Leaky Integrate-and-Fire (LIF) neuron, which integrates the signal and encodes it into spiking activity. This spiking activity is passed to a decoder layer to reconstruct the original heart rate signal and to a spiking neural state machine. The state machine, depending on the specific implementation, extracts additional information about the status of different frequency bands within the signal.

The schematic diagram describing the main components of the proposed system is depicted in Fig. 1. Multimodal biosignals are processed by a filter-bank of band-pass filters which isolate the frequency components relevant to HR detection. The filter outputs are subsequently routed to two parallel computational pathways: a *decoder* for monitoring and detecting heart rate in Beats per Minute (bpm), and a NSM for detecting state transitions and trends in HR dynamics. Even though we chose to use only four filters for the corresponding HR bands, the architecture is modular and scalable, allowing the use of additional filters and nodes in the processing modules, to increase the frequency resolution and enable finer-grained detection of bio signal zones.

The decoder performs an on-line estimate of the heart rate, by processing the filter outputs on-line with a spiking neural network, which maps the activity of its neurons to discrete HR values. In parallel, the filter outputs are processed by the NSM, which detects and manages HR state transitions robustly even under noisy and dynamic conditions, by adopting the same strategies used by cortical circuits in animal brains. Namely, population coding, averaging, and recurrence under Excitatory-Inhibitory (EI) balance (see “The Neural State Machine architecture”).

We validated the architecture and verified its expected performance by implementing it in hardware, on a mixed-signal Dynamic Neuromorphic Asynchronous Processor (DYNAP-SE) chip comprising four cores of 256 silicon neurons each, with 64 configurable dynamic synapse circuits/neuron: the DYNAP-SE^15^. To assess the system’s robustness, we conducted repeated measurements across multiple independent initializations on different days, evaluating it’s resilience to parameter variations. The consistent performance observed across multiple trials confirms that noise and variability does not significantly impact the system’s functionality.

We tested the performance of three distinct NSM architectures to manage HR band selection in dynamic and noisy environments. The first architecture implements a soft Winner-Take-All (WTA) network which performs competitive selection among populations of excitatory neuron^46^. The WTA selects the most active HR band currently being measured. As the WTA network dynamics are set to relatively be slow compared to measurement artifacts (i.e., with time constants of hundreds of milliseconds) the HR zone is encoded reliably and in a stable manner. This approach offers a fast and reliable method for the section of the current state.

The second architecture, denoted as Nearest Neighbors Neural State Machine (nnNSM), incorporates additional disinhibition dynamics among nearest neighbor populations to allow state transitions only between adjacent states. This design reduces sensitivity to large HR fluctuations, ensuring smoother and more gradual state transitions while improving noise resilience.

The third architecture, denoted as Monotonic Neural State Machine (monoNSM), enforces a unidirectional progression through HR bands. By implementing hierarchical routing with lateral inhibitory pathways, the monoNSM prevents state overlap and stabilizes transitions, making it well-suited for detecting monotonic trends in heart rate over long periods of time (e.g., multiple hours).

We evaluated the system performance using both synthetic data (see “Synthetic dataset”) and two real-world datasets: the BIDMC and WristPPG (see “Biomedical datasets”). We used synthetic data to assess the network’s robustness under varying noise levels in a controlled environment. At the same time, the real-world datasets were used to test its behavior under real-world noisy conditions. On clean BIDMC data, the architectures achieved near-ground-truth accuracy in HR band decoding (see Figs. 3f, 4e). The WristPPG dataset posed greater challenges due to motion-induced noise. To further improve performance, we integrated accelerometer data in the architecture, demonstrating that it effectively mitigated motion artifacts in the WristPPG dataset and enhanced the NSM’s state detection (see Fig. 5b, c)

**Fig. 2:**
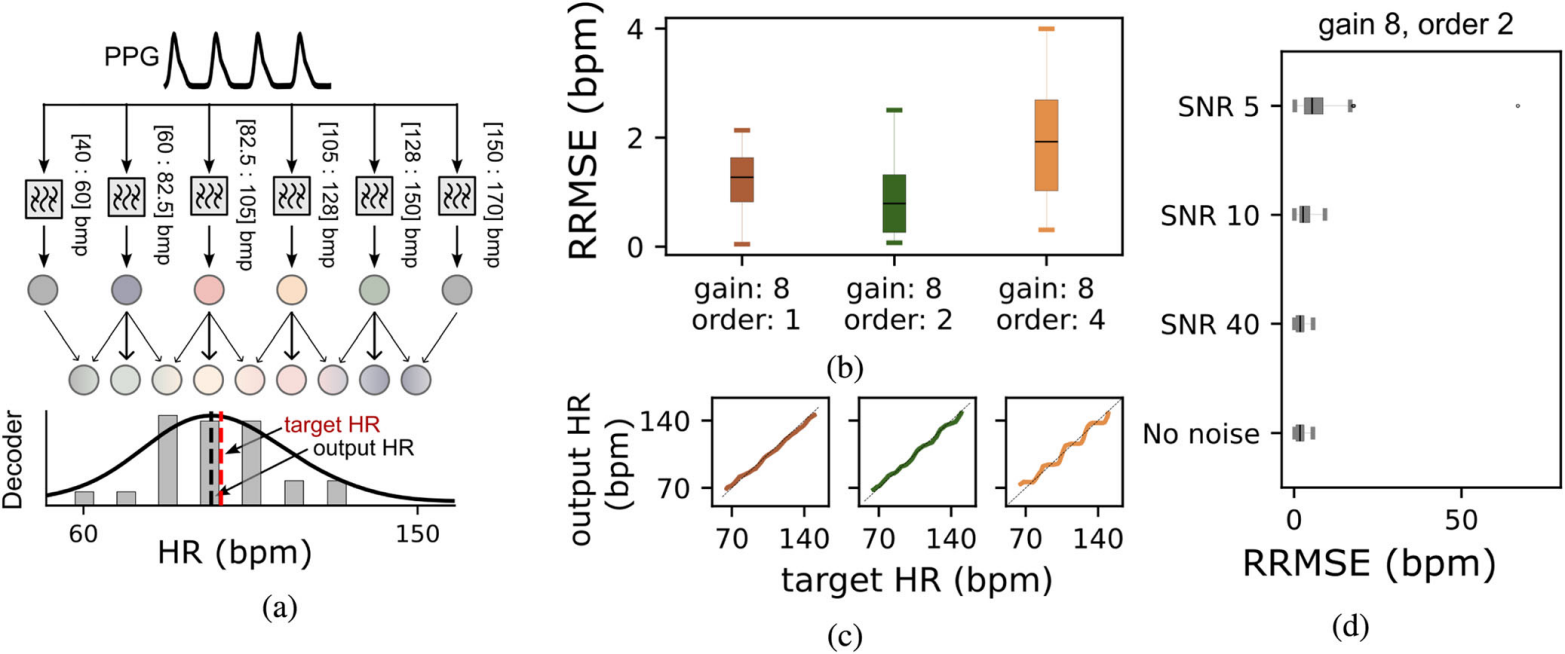
Schematic of the neuromorphic system. **a** Biosignals are processed by a bank of bandpass filters, including two boundary filters to avoid decoding errors at signal edges. Each filter output connects to a Leaky Integrate-and-Fire (LIF) neuron, which encodes signals into spikes. A second processing layer integrates spikes from each neuron and its neighbors to compute a Gaussian-weighted distribution of firing rates, improving Heart Rate (HR) estimation accuracy. **b** Performance of the system evaluated as Relative Root Mean Square Error (RRMSE) between the target and decoded HR. **c** Decoded and target HR for each pass-band filter order. **d** System’s performance on signals corrupted by white noise was evaluated using the RRMSE between the target and decoded HR.

Figure 2a shows the structure and performance of the decoder designed for HR estimation. To address edge effects, the filter bank was extended with two additional boundary filters which ensure continuous and accurate HR decoding by stabilizing the outputs at the edges of the signal. Each filter output is connected to a LIF neuron, which converts the filtered signals into spike trains. These spike trains are further processed by an additional neural layer on the DYNAP-SE. This layer incorporates connections from the corresponding LIF neuron and its neighbors to approximate a Gaussian-weighted distribution of firing rates. To calculate a more accurate estimate of the HR, we smooth the decoder activity with a Gaussian kernel and detect it’s maximum (see solid line of Fig. 2a. This operation is done off-line and is not implemented on-chip. Figure 2b shows the performance of the decoder evaluated in terms of RRMSE between the target HR, defined by the synthetic data, and the system’s decoded output. We can fine-tune the decoder performance by adjusting the gain of the filters and their order. The best results are obtained by setting a gain of the decoder filter bank to eight, and their filter order to two. With these settings the decoder achieves an RRMSE of about 1 bpm, demonstrating highly accurate HR estimation, despite the simplicity of the network structure. Interestingly, the best performance is achieved with low-order filters, which allow slight frequency overlap with neighboring bands (see Fig. 2c) reducing the “within-band” plateau. The overlap in frequency bands improves the Gaussian-weighted firing rate distribution, enhancing the system’s robustness and accuracy in decoding HR. The results highlight how tuning filter characteristics–particularly the balance between sharp band isolation and slight leakage–can significantly impact decoding performance. Given that physiological HR variations naturally fluctuate within small margins, achieving higher precision is not critical for these applications. We evaluated the system’s performance on signals corrupted by white noise using the RRMSE between the target and decoded HR (see Fig. 2d). Although low Signal to Noise Ratio (SNR) values negatively impact reconstruction accuracy, the system demonstrates robust performance overall. We additionally performed a power analysis of the on-chip decoder network for different configurations based on the circuit equations (see “The DYNAP-SE neuromorphic hardware”). Each configuration consists of a population of 13 neurons with varying mean firing rates observed during operation. The results for five-second trial sessions are as follows: *order 1 and gain 8*: With a mean firing rate of 32.67 Hz, the estimated power consumption was 20.3 *μ*W, leading to an energy consumption of 101.5 *μ*J. *order 2 and gain 8*: With a mean firing rate of 21.1 Hz, the estimated power consumption was 13.1 *μ*W, resulting in an energy consumption of 65.5 *μ*J. *order 4 and gain 8*: With a mean firing rate of 17.9 Hz, the estimated power consumption was 11.1 *μ*W, corresponding to an energy consumption of 55.5 *μ*J.

**Fig. 3:**
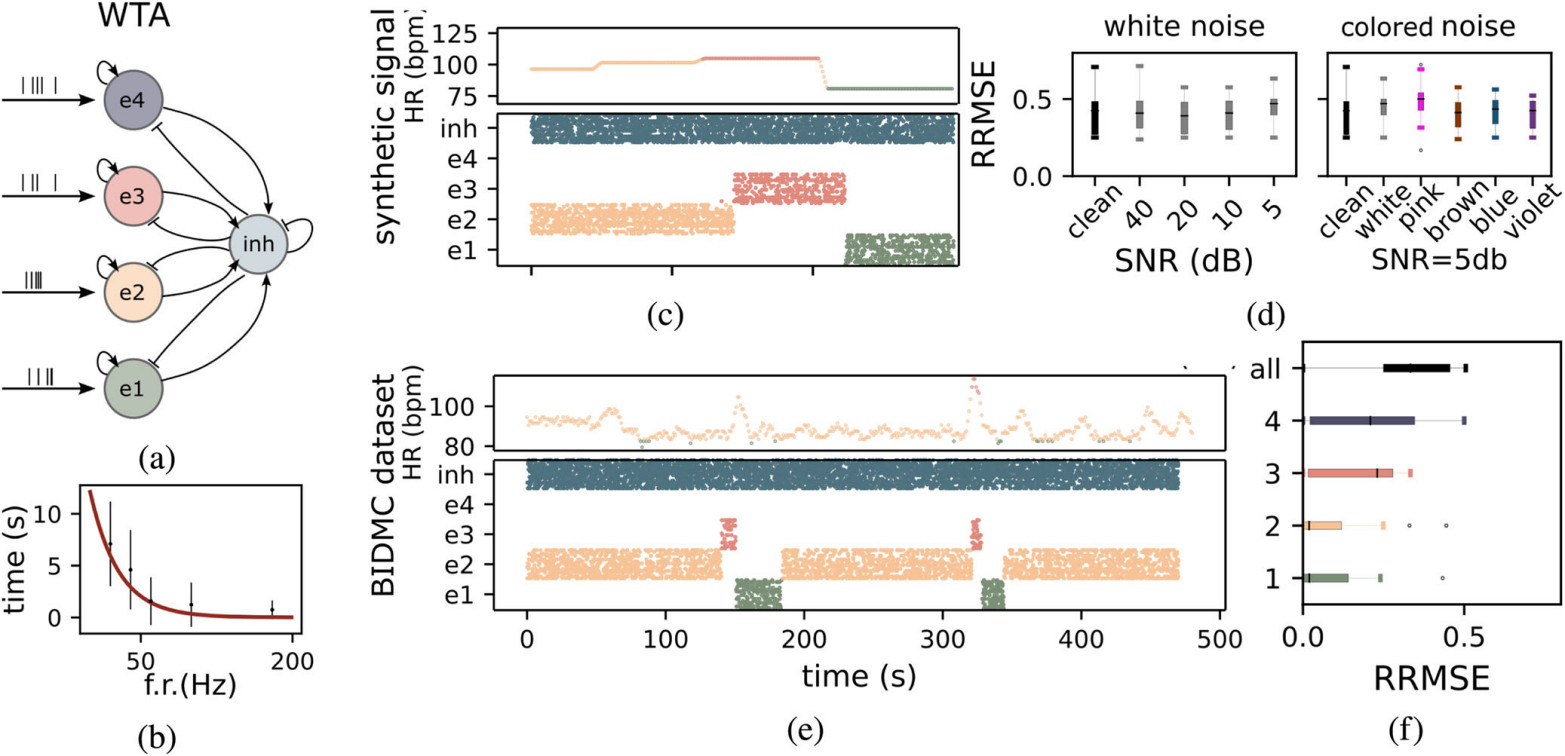
Spiking activity and performance evaluation in the WTA architecture. **a** Architecture of the WTA network used for heart rate band selection. Excitatory neuron groups (e1-e4) are linked to specific heart rate bands, while an inhibitory population (inh) regulates competition among bands. **b** Network transition time between the two states versus the second state input firing rate. The transition is not instantaneous but depends on the strength and duration of the external input. Stronger input signals lead to faster transitions, while weaker inputs require a longer duration to induce a state change. **c** *Bottom:* network spiking activity example, with transitions between bands highlighting dynamic heart rate changes when presented with a synthetic dataset. *Top:* corresponding ground truth Heart Rate (HR) in bpm. **d** *Left:* Relative Root Mean Square Error (RRMSE) across different Signal to Noise Ratio (SNR) for the synthetic dataset. *Right:* RRMSE for clean signals and signals contaminated by various types of noise (white, pink, brown, blue, violet). **e** *Bottom:* network spiking activity example when presented with a BIDMC PPG data. *Top:* corresponding ground truth HR. **f** RRMSE for heart rate estimation of each and all bands.

The soft WTA network receives in input the spikes generated by the decoder and exhibits spiking dynamics which lead to clear state transitions, as illustrated in Fig. 3. The network, evaluated using both synthetic and BIDMC datasets, can handle diverse input distributions reliably. The raster plots of Fig. 3c, e depict the typical dynamics of the WTA in response to the spiking inputs. Over time, the system exhibits a progression of discrete state transitions, with each state (e1, e2, e3, e4) corresponding to a specific heart rate band. These transitions are not instantaneous but depend on the strength and duration of the external input, as well as the network’s integration time scale. Thanks to its smooth dynamics, the network reliably switches between the different bands, without being affected by potential transients or glitches in the input signals. To determine the transition time between two states we let the network settle into one stable state and then stimulated a different band with varying firing rates. The transition to a new state does not occur immediately but requires the new input to be sustained for a sufficient duration. We measured the transition time required for the stimulated population’s firing rate to surpass the previously active population. We show that stronger input signals lead to faster transitions, while weaker inputs require a longer duration to trigger state changes, demonstrating that the system adapts to more pronounced stimuli efficiently (see Fig. 3b). To quantitatively evaluate the network’s performance in encoding state transitions, the RRMSE was computed by comparing the network’s dynamics against the ground truth. This evaluation was performed on synthetic data to study the impact of varying levels of white noise and different noise spectral properties (white, pink, brown, blue, and violet). As shown in Fig. 3d, increasing levels of white noise led to a slight increase in RRMSE, with performance remaining robust. Additionally, the figure explores the effects of different noise types at an SNR of 5 dB. Pink noise, characterized by high power in the lower frequency spectrum, exerted the most significant influence on network dynamics, resulting in higher RRMSE values compared to other noise types. This effect is attributed to pink noise amplifying higher harmonics in the signal, particularly in the higher frequency bands. For the real BIDMC dataset, the measured HR was assumed to represent the ground truth for performance comparison. Figure 3f shows the RRMSE values for individual states as well as the overall system performance. Lower RRMSE values observed for lower states (e1 and e2) indicate greater robustness to noise, while higher RRMSE values for the upper states (e3 and e4) suggest increased sensitivity to disturbances. This increased sensitivity in higher states is likely due to a higher harmonics phenomenon in these bands.

**Fig. 4:**
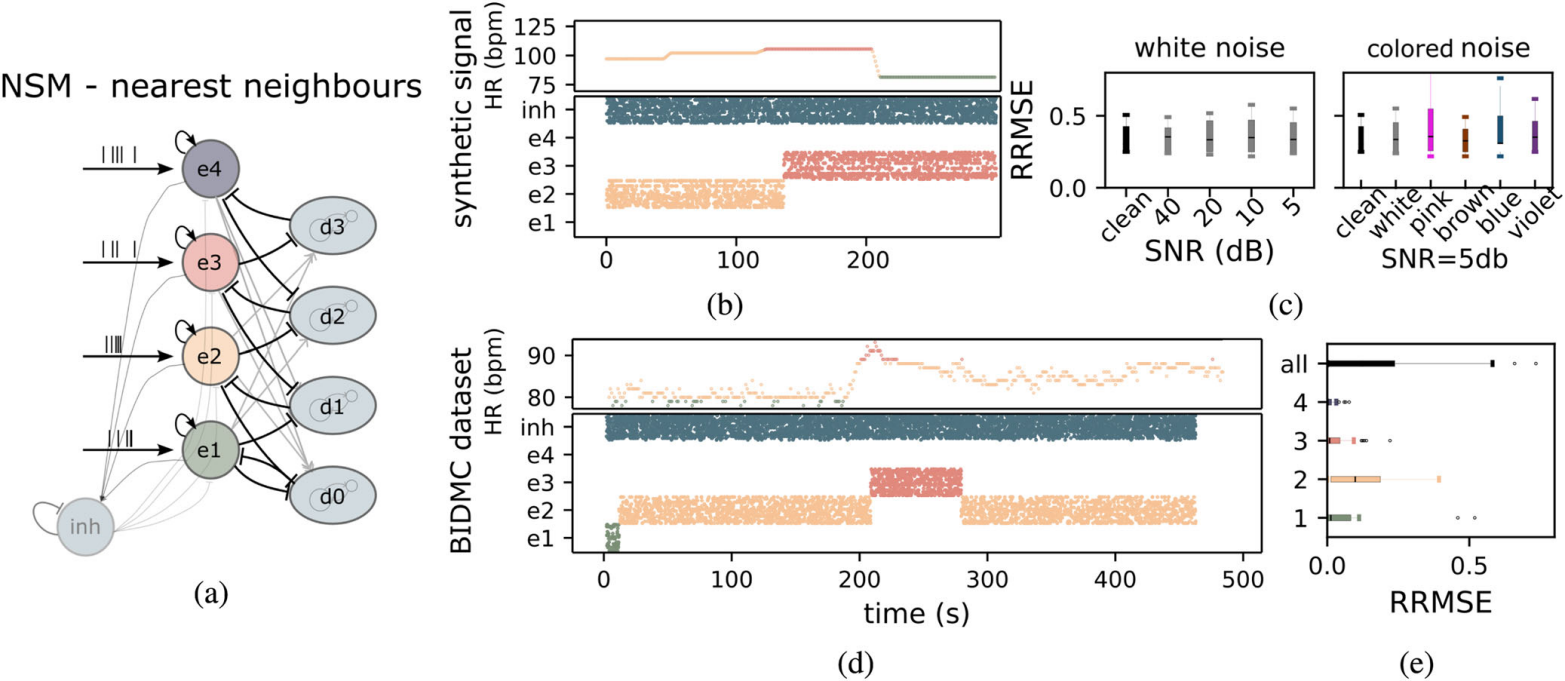
Spiking Activity and Performance Evaluation of the Nearest Neighbors Neural State Machine (nnNSM). **a** Architecture of the nnNSM used for heart rate band selection. This modified Winner-Take-All (WTA) design incorporates disinhibition populations, enabling smooth transitions between heart rate bands. **b** *Bottom:* network spiking activity example when presented with a synthetic dataset. *Top:* corresponding ground truth Heart Rate (HR) in beats per minute. nnNSM network does not transit between non-adjacent states. **c** *Left:* Relative Root Mean Square Error (RRMSE) across different Signal to Noise Ratio (SNR) for the synthetic dataset. *Right:* RRMSE for clean signals and signals contaminated by various types of noise (white, pink, brown, blue, violet). **d** *Bottom:* network spiking activity example when processing BIDMC PPG data. *Top:* ground truth heart rate (in bpm), with the system accurately reflecting variations in HR. **e** RRMSE of heart rate estimation for each band and the entire system (all).

The nnNSM was designed to allow transitions only between adjacent states. This is achieved by including a biologically realistic dis-inhibition mechanisms, which ensures smoother and more controlled state transitions^47^. As illustrated in Fig. 4a this design balances excitatory and disinhibitory populations, enabling effective monotonic progression between heart rate bands (e1, e2, e3, e4). By enforcing transitions only among nearest neighbors, the nnNSM avoids abrupt jumps, addressing a key limitation of the WTA approach. This results in higher robustness to noise and more accurate HR estimation, particularly in the upper bands (e3, e4), where the WTA exhibits greater sensitivity to noise. The network raster plots show distinct spiking activity for excitatory populations (e1, e2, e3, e4) and disinhibitory populations (d0, d1, d2, d3) under the influence of input stimuli. Each excitatory state represents a heart rate band, with transitions occurring only between adjacent bands, enhancing noise resilience and preventing abrupt HR estimation changes. The temporal evolution of network activity demonstrates smooth state progression in response to varying input, Fig. 4b. Performance metrics highlight the network’s resilience across various noise conditions. Under synthetic data with white noise, RRMSE increased only slightly as SNR decreased to 5 dB, demonstrating robustness. Colored noise, particularly pink noise with high energy in lower frequencies, had the most significant impact. However, the mean RRMSE remained consistent across noise types, with a slightly higher standard deviation for pink noise (see Fig. 4c). When applied to the real BIDMC dataset, the nnNSM achieved lower RRMSE values than the WTA network, especially in edge states (e1, e4), as shown in Fig. 4e. This improvement is attributed to the monotonic design, which reduces errors by activating higher bands only under appropriate input conditions. The system also successfully addressed high-frequency noise in real-world data, maintaining accurate decoding of HR dynamics.

The estimated power consumption of the neuromorphic long-term state detection system was calculated using the equation described in “The DYNAP-SE neuromorphic hardware”. For this estimation, a population of 16 neurons was considered for both the WTA and nnNSM networks. The mean firing rates observed during operation were 25 ± 2 Hz for WTA neurons and 30 ± 7 Hz for nnNSM neurons. Based on these values, the total power consumption of the network was estimated to be approximately 61.6 *μ*W. Over the course of a 470 s trial, the cumulative energy consumption amounted to 29 mJ.

**Fig. 5:**
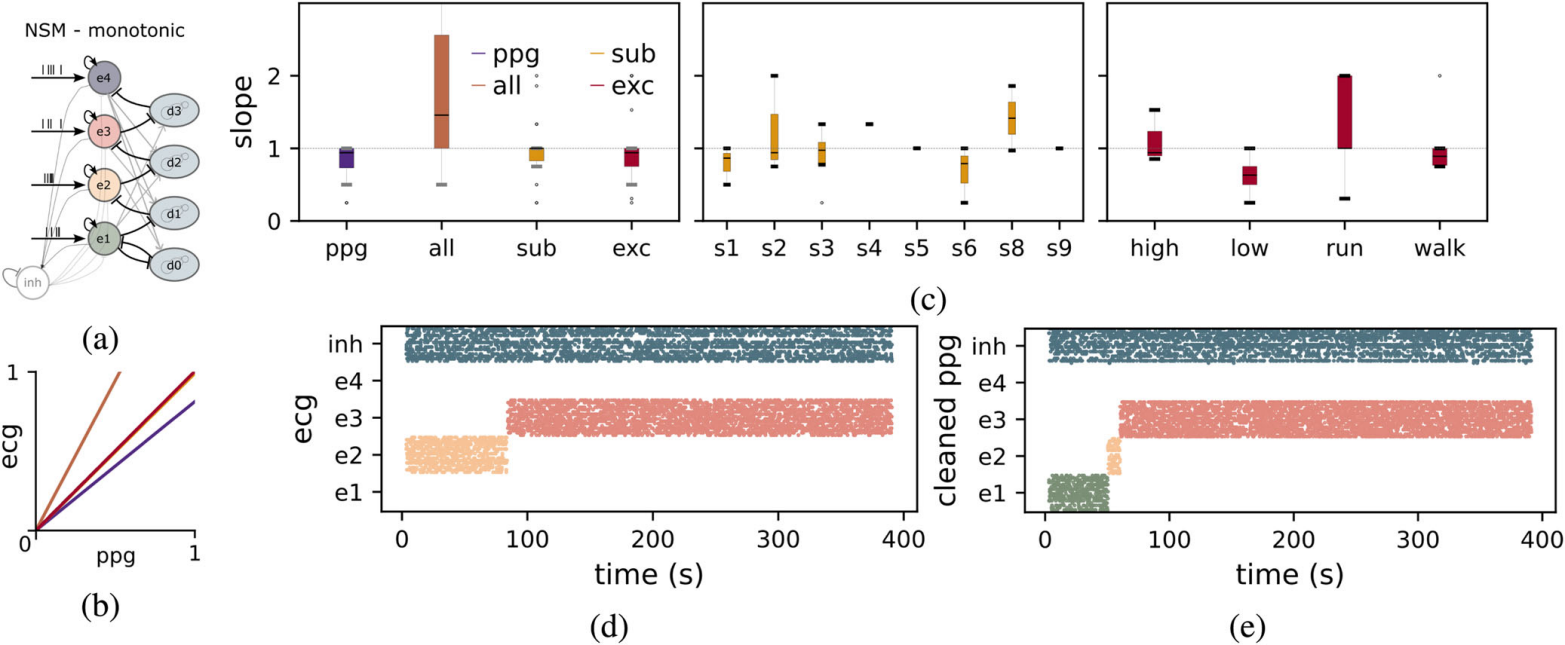
Neural state machine for detecting monotonic changes in heart rate amidst motion artifacts. **a** Illustrates the competitive interactions between excitatory populations (e1 to e4) and disinhibitory populations (d0 to d3), which facilitate state transitions and noise suppression. **b** Fitted slope of firing rates for the Leaky Integrate-and-Fire (LIF) firing rate output from the Electrocardiography (ECG) signal versus different Photoplethysmography (PPG)-Accelerometers signal combinations optimization: raw PPG (purple), optimized over all samples (orange), subject-specific optimization (yellow), and exercise-specific optimization (red). **c** *Left:* slope distribution for each signal optimization. (raw PPG signal, all dataset, subject-specific and exercise specific). **Center:** slope distribution for each subject (s1-s9) and *Right:* slope distribution for each activity type (high resistance, low resistance, running, and walking). **d** Raster plots of raw ECG-derived states (left) and **e** artifact-cleaned PPG-derived states (right). The improved separation and stability of states in the cleaned PPG signal highlight the effectiveness of the optimization in mitigating motion artifacts, enabling robust heart rate monitoring during dynamic activities.

Finally, the monoNSM illustrated in Fig. 5a, demonstrates a robust architecture designed to ensure transitions occur progressively toward higher HR zones. This approach is particularly useful for monitoring activities where gradual increases in HR over extended periods of time (e.g., hours) are expected, such as sports performance or specific pathological scenarios.

Using the Wrist dataset, which includes PPG, ECG, and accelerometer data collected during various physical activities (e.g., walking, running, high- and low-resistance cycling), we analyzed the monoNSM’s ability to process noisy input signals. Since the dataset lacked a precise HR ground truth, ECG signals were used as a surrogate. In real-world applications where ECG is not available, alternative strategies for artifact elimination could be employed, such as optimizing the linear combination based on expected signal characteristics or utilizing unsupervised learning approaches trained on large datasets. These alternatives could offer flexibility when ground truth data is unavailable.

PPG signals, which were heavily affected by motion artifacts, were cleaned using accelerometer data to align them with ECG-derived firing rates. To address mootion artifacts, accelerometer data was used as a reference, with ECG recordings serving as a surrogate ground truth in the absence of precise HR data. Signals were preprocessed as described in “Signal preprocessing”, and a linear combination of four PPG signals and 12 filtered accelerometer signals (four filters per axis) was optimized using Nelder-Mead algorithm^48^ to minimize the RRMSE between LIF firing rates and ECG-derived rates. Gradient-based methods were avoided due to the overly smooth gradient landscape. Despite being simpler than advanced techniques like deep neural networks this approach yielded state-of-the-art results. Figure 5c, b highlights the LIF neuron firing rates for various conditions: unoptimized PPG signals (purple), generalized optimization (orange), subject-specific optimization (yellow), and active-specific optimization (red). Tailored optimizations, either by subject or activity, significantly improved performance, highlighting the importance of calibration for specific users or exercise types. In this context, exercise-type-specific optimization offers flexibility for real-world applications, allowing for dynamic parameter adaptation based on the ongoing activity, thus improving artifact elimination in the absence of precise ECG signals. Figure 5e, d shows raster plots comparing raw ECG-derived signals and artifact-cleaned PPG signals over time. The cleaned signals display improved separation of states, indicating successful artifact removal and accurate transition control by the monoNSM. The smooth transitions between excitatory states reflect the system’s stability and its capacity to handle dynamic physiological inputs under noise-induced conditions. Overall, the monoNSM system is able to effectively process noisy input data while maintaining high-resolution monotonic state transitions across diverse contexts, demonstrating the potential to adapt to diverse real-world scenarios even in the absence of ECG signals, provided with suitable dynamic parameter adaptation strategies.

## Discussion

Our work introduces a fundamentally different approach to HR detection by operating computations entirely at the neural network hardware level, eliminating the need for microprocessors to execute software-based algorithms. By leveraging mixed-signal neuromorphic circuits to implement neural computational primitives, such as winner-take-all mechanisms and state-dependent selection directly on-chip, the system achieves robust performance with significantly reduced power consumption. A notable strength is its ability to clean signals at the hardware level using inhibitory synapses between LIF neurons to subtract noise and artifacts, bypassing software-driven noise reduction techniques and providing a low-power, cost-effective solution.

Additionally, we propose a novel approach for motion artifact removal that integrates accelerometer data directly into the analog signal processing pipeline. By performing subtraction between bandpass filter outputs in each filter bank, motion-induced noise is effectively isolated and removed at the circuit level. Inspired by, and compatible with, the SPAIC (signal-to-spike converter for analog AI computation) analog front-end chip^49^, this approach achieves highly efficient signal processing. Each channel in SPAIC consists of a Low-Noise Amplifier (LNA), a flipped voltage follower-based bandpass filter, and a Programmable Gain Amplifier (PGA), forming a modular and low-power signal conditioning block. With a bias current of 200nA per amplifier, a system with four filter banks per PPG signal and per accelerometer consumes approximately 11.52 *μ*W per bandpass filter bank. This inherently low-power design makes the proposed artifact removal approach ideal for always-on wearable devices.

The filter bank design ensures scalability and flexibility, with the current implementation of four filters achieving effective performance. The analog-to-spike conversion is performed using Pulse Frequency Modulation (PFM) by LIF neurons. We demonstrated how the system’s robustness to parameter variations allows it to maintain stable performance. This stability allowed us to reducing the number of output spikes, by selecting a higher spiking threshold, and consequently to further reduce power consumption. Additional filters can be added to improve resolution with minimal complexity. As shown in Fig. 2, the decoder isolates HR frequency bands and mitigates edge effects using two boundary filters. The processed signals are translated into a Gaussian distribution of firing rates, achieving an RRMSE of 1 bpm under optimal conditions. However, the system already achieves high accuracy with just four low-order bandpass filters. Increasing the number of filters is not necessary, given that for all practical purposes higher precision is not necessary, also due to the fact that physiological HR variations typically fluctuate within narrow ranges. In addition, the usage of low-order filter improves precision reducing the “within-band” plateau allowing also for higher power efficiency and reduced latency, making the system ideal for real-time wearable applications. For other applications requiring finer temporal or frequency resolution, additional filters could be easily incorporated to improve discrimination. While this may benefit broader HR range estimation or more granular frequency analysis, it introduces trade-offs in terms of power and computational complexity. In contrast, our approach balances efficiency and accuracy, achieving an RRMSE of approximately one bpm, which compares favorably to state-of-the-art methods typically reporting errors around four bpm^50^. The modular design nature of the architecture minimizes computational redundancy and ensures efficient, real-time processing.

The WTA mechanism is highly effective in identifying general signal zones (e.g., low, medium-low, medium-high, high) for applications like sports monitoring or physiological analysis, where exact HR values are unnecessary. Its lightweight, hardware-efficient design ensures reliable performance under various noise conditions. The system maintains low RRMSE even at low SNR (e.g., 5 dB) and handles transient artifacts by reacting only to persistent signals. While pink noise amplifies intrinsic higher harmonics in higher bands, the system’s stability remains uncompromised, with challenges like back-and-forth state switching mitigated by adding inhibitory mechanisms. With a switching time of 5 seconds, the WTA is well-suited for stable signals, though faster responsiveness (1-1.5 seconds) can be achieved for dynamic scenarios by increasing firing rates.

The nnNSM addresses the limitations of the WTA approach by enabling smooth, monotonic transitions between adjacent heart rate bands, enhancing noise robustness and preventing abrupt HR changes. Under synthetic noise conditions, the nnNSM demonstrated resilience across all noise types, with minimal variability in RRMSE values. Testing with the BIDMC dataset showed that nnNSM outperformed WTA, particularly in edge states (e1, e4), achieving lower RRMSE values and greater resilience to noise. Its monotonic behavior supports long-term physiological monitoring, such as tracking gradual HR changes in dementia patients, where low-power, stable operation is essential. Hardware-level state preservation further enhances its reliability, with tests confirming state stability for over 9 hours during power interruptions. Transitions occur within approximately two seconds, making the nnNSM a robust and responsive solution for real-time applications.

The monoNSM represents a significant advancement by enforcing monotonic transitions toward higher HR zones. This is particularly effective for applications requiring gradual HR changes over long time periods, such as sports performance monitoring or real-time tracking of pathological states. By exploiting dis-inhibition mechanisms between states, the monoNSM ensures smooth transitions, avoiding abrupt changes and enhancing robustness. Evaluations using the Wrist dataset demonstrated its ability to handle noise and variability during activities of varying intensity levels. Personalized and activity-specific optimizations significantly improved performance, especially for motion-intensive activities like running. This adaptability highlights its potential for wearable devices supporting user-specific calibration or activity-based modes. Unlike offline, computationally intensive methods, the monoNSM supports real-time hardware implementations, enabling efficient operation in diverse, noisy environments. However, the small dataset used in this study limits generalizability, suggesting that future work should focus on expanding datasets to further assess robustness and scalability across broader applications.

Monitoring HR changes over time is crucial for understanding physiological and pathological states and optimizing performance during physical activities. Neuromorphic networks, including NSM and WTA models, provide efficient, hardware-compatible solutions for tracking activity zones in real time. These approaches excel in scenarios requiring detection of monotonic HR changes, offering insights into exercise intensity, recovery phases, or stress levels. Their adaptability and low-power operation make them ideal for wearable devices supporting personalized monitoring and healthcare interventions. All of the required processing, occurring over multiple timescales can be seamlessly performed on the same device, integrating both front-end analog processing and mixed-signal neural network modules.

Furthermore, although the basic operations of NSMs are akin to digital finite state machines, they support additional computational features and provide additional advantages compared to their simple digital counterparts, including the ability to process asynchronous events directly, to operate in a stochastic/probabilistic mode, the ability to express complex temporal dynamics through E-I balanced network interactions, and to exhibit fault tolerance and robustness to noise. An important consideration in neuromorphic circuits is the influence of non-ideal device behavior, such as transistor mismatch. Rather than viewing this as a limitation, our approach actively leverages mismatch to enhance robustness. In the E-I balanced networks that are part of the NSMs, device mismatch prevents unwanted synchronous oscillatory behavior, thereby promoting more stable and diverse neuronal activity. The brain-inspired population coding strategy used in these networks mitigates the impact of individual neuron variability, ensuring robustness at the level of the overall functionality of the system. The strong competitive dynamics of the WTA mechanism further suppress device mismatch effects increasing reliability in the selection of relevant signals. We validated the system’s resilience by measuring consistent performance across repeated experiments from multiple independent chip initializations on different days, confirming that noise and variability does not compromise the system’s reliability. While chip-to-chip variability might require initial recalibration of circuit parameters, this is compatible with analogous subject-to-subject calibration routines in biomedical devices and does not affect the underlying computational principles.

The results presented demonstrate the system’s ability to adapt to noise in input signals and variability in the architecture’s computing element, as is the case for ultra-low-power analog neuromorphic circuits, while maintaining accuracy and robustness. The successful validation of this architectures with an existing neuromorphic research platform demonstrates that it can be mapped onto dedicated hardware for realizing a versatile solution for continuous, long-term health monitoring.

Power consumption is a key consideration in always-on signal-processing systems, especially for wearable applications that require long-term continuous operation for real-time physiological monitoring. Minimizing energy usage is essential to extending battery life, reducing recharging frequency, and ensuring long-term functionality. To assess overall consumption, we estimated the power required when both the decoder for HR estimation and the nnNSM are active for long-term analysis. If the order 2, gain 8 decoder operates continuously alongside the WTA and nnNSM networks, the total power consumption of the neuromorphic system would increase. Based on circuit-level estimations, the WTA and nnNSM networks together consume 61.6 *μ*W, while the decoder adds 13.1 *μ*W, bringing the total to 74.7 *μ*W. Over the course of an hour, this results in an energy consumption of 268.9 mJ. Considering a standard 3.7 V, 100 mAh lithium-ion battery commonly used in wearable devices, which stores approximately 1332 J (assuming full capacity and ideal discharge), this part of the system could theoretically run continuously for months (6̃) before requiring a recharge. This level of efficiency significantly outperforms equivalent deep learning-based solutions, optimized for embedded integration^36^, which consumes 47.65 mJ per inference (every 10 seconds) in its most accurate model, making neuromorphic processing a superior approach for ultra-low-power, always-on monitoring. Even though this estimation does not take into account the power consumption of the PPG sensors, for both neuromorphic-based and deep-network based approaches, it underscores the feasibility of the neuromorphic approach for continuous, low-power physiological monitoring in wearable applications. The ultra-low power requirements enable extended operation without frequent recharging, making it particularly suitable for medical wearables, elderly care, and remote health monitoring, where maintenance and battery replacements must be minimized.

## Methods

### Synthetic dataset

A synthetically generated PPG signal has been developed to provide precise control over the signal-to-noise ratio and the dynamics of heartbeats per minute. The signal was implemented approximating a single PPG pulse with two Gaussian functions^51^ and applying the circular motion principle to distribute PPG pulses over time. The overall dynamics can be described as:

1 $$\left\{\begin{array}{l}x\left(t\right)=cos\left(\omega \left(t-{t}_{0}\right)-\pi \right)\quad \\ y\left(t\right)=cos\left(\omega \left(t-{t}_{0}\right)-\pi \right)\quad \\ z\left(t\right)=\mathop{\sum }\limits_{i = 1}^{2}{a}_{i}{e}^{-\frac{{\left(\theta \left(t\right)-\theta i\left(t\right)\right)}^{2}}{2{b}_{i}^{2}}}\quad \end{array}\right.$$

where *x*(*t*) and *y*(*t*) describe a cyclic dynamics and *ω* = 2*πν* the angular velocity. *z*(*t*) is modeled as the sum of two Gaussians, representing the amplitude of the synthetic PPG signal, whit *a*_*i*_ representing their peak values, 2*b*_*i*_ their *σ* and $\theta (t)=atan2\left(y(t),x(t)\right)$ as the angle in polar coordinates. The constants *t*_0_ and *θ*_*i*_(*t*) define the initial conditions. Used parameters are reported in Table 1.

**Table 1:** Synthetic signal parameters

a_1_	a_2_	b_1_	b_2_	*θ*_1_	θ_2_	t_0_
0.19 ± 0.02	0.07 ± 0.01	0.42 ± 0.01	0.5 ± 0.4	-0.7 ± 0.1	0.4 ± 0.1	0.403 ± 0.008 s

To determine the parameters of the synthetic PPG waveform, 10 events were randomly selected from a real PPG sample in the BIDMS dataset (trial bidmc01). A PPG pulse was defined as the data points between the midpoints of two consecutive valleys at the boundaries of a PPG event. The model was then fitted to each of the 10 selected events, and the final parameters were calculated as the averages of the fits: All experiments in this study involving synthetic PPG signals are based on the parameters reported above.

#### Noise

To simulate a continuous signal with added noise at varying SNR in decibels and to incorporate additional noise of specific colors, a systematic approach is followed. Based on the desired SNR in dB, the required noise power is determined using the relationship between signal power *P*_*signal*_ and noise power *P*_*noise*_, so that

$${P}_{noise}={P}_{signal}* 1{0}^{-SNR/10}$$

White noise, characterized by a flat power spectrum, is generated as a random Gaussian signal with zero mean and a variance *P*_*noise*_. This white noise is then added to the original signal, producing a noisy version of the signal for each specified SNR value. The procedure is repeated iteratively for multiple SNR levels, enabling the generation of noisy signals with varying degrees of noise intensity. To introduce additional colored noise, white noise is filtered to modify its spectral density. Finally, the colored noise is added to the previously generated noisy signals, producing a final signal that incorporates both white noise and colored noise.

### Biomedical datasets

To test the networks implemented, we selected real-world datasets of PPG signals, choosing two complementary datasets to address different scenarios. The first dataset (BIDMC) provides clean measurements under controlled conditions, including a reliable ground truth heart rate. The second dataset (WristPPG) captures signals during dynamic activities with varying heartbeats. For this latter dataset, we developed a hardware-aware cleaning method leveraging accelerometer signals to mitigate motion artifacts (see “Motion artifact mitigation”).

To evaluate our model, we utilized a publicly available dataset from the IEEE Dataport, which contains multimodal recordings of EEG, PPG, blood pressure, and respiratory signals^52^. These recordings were collected from 53 subjects under various physiological conditions, including resting states and activities designed to induce changes in cardiovascular and respiratory dynamics, such as controlled breathing and postural changes. Each session lasted approximately 8 minutes, with all signals sampled at 125 Hz. Importantly, the signals provided in this dataset are relatively clean, as the controlled conditions minimize movement artifacts, reducing noise and simplifying signal processing. Additionally, the dataset provides a ground truth heart rate measurement sampled at 1 Hz, allowing for reliable validation of heart rate estimation methods.

To evaluate our model, we utilized a publicly available dataset containing left wrist PPG, chest ECG recordings, and motion data from accelerometers and gyroscopes^53^. These recordings were collected while participants engaged in physical activities using an indoor treadmill and exercise bike. The dataset captures various exercises, including walking, light jogging/running on a treadmill, and cycling at low and high resistance, with each activity lasting up to 10 minutes. The dataset includes recordings from eight participants, with most participants performing each activity for 4 to 6 minutes. All signals were sampled at a frequency of 256 Hz, and the ECG data underwent preprocessing with a 50 Hz notch filter to mitigate mains power interference. The interest of this dataset lies in the involvement of the subjects in dynamic activities, allowing us to observe significant variations in heart rate. On the other hand, these activities introduce substantial motion-related noise in the sensor data, posing a challenge for accurate signal analysis. It is important to note that the dataset lacks a ground truth reference for heart rate, further emphasizing the complexity of deriving reliable physiological metrics from these recordings.

### Signal preprocessing

The signal preprocessing follows an energy-based approach for signal-to-spike conversion^20,54^. The procedure consists of two main stages: bandpass filtering and Leaky Integrate-and-Fire (LIF) neuron encoding^55^. Initially, each input channel is processed through a series of 4 bandpass filters to isolate frequency components corresponding to different heart rate bands, using fourth-order Butterworth filters. After filtering, the signal undergoes full-wave rectification, ensuring that the entire waveform is positive. The rectified signal is then amplified before being injected as a time-varying current into a Leaky Integrate-and-Fire (LIF) neuron, which integrates the current and encodes it as spiking activity. This encoding stage allows the continuous signal to be represented in discrete spikes, which can then be processed by subsequent layers of the neuromorphic system.

**Fig. 6:**
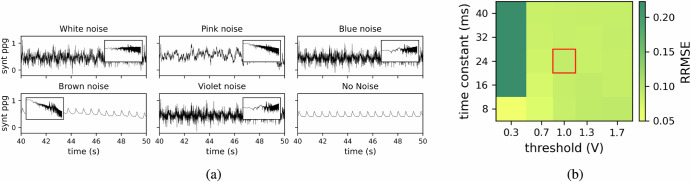
Impact of noise and LIF neuron parameters on signal reconstruction accuracy. **a** Examples of synthetic signals with different types of noise for a Signal to Noise Ratio (SNR)=10. Synthetic signals with white noise (flat power spectrum), pink noise (power inversely proportional to frequency), blue noise (power directly proportional to frequency), brown noise (power inversely proportional to the square of the frequency), violet noise (power proportional to the square of the frequency), and the noise-free signal for comparison. Insets display the corresponding power spectral density for each noise type, highlighting the spectral characteristics. **b** Impact of Leaky Integrate-and-Fire (LIF) neuron parameters on decoder reconstruction accuracy. The heatmap illustrates the Relative Root Mean Square Error (RRMSE) of decoder reconstruction for different preprocessing parameters of LIF neurons. It represents the RRMSE between the reconstructed Heart Rate (HR) and the target HR for a synthetic signal. The decoder network processes input signals converted into spike trains by LIF neurons, with varying time constants and spiking thresholds.

Such LIF neurons are governed by two key parameters: the neuron time constant *τ* and the neuronal spiking threshold *V*_*thr*_. Throughout this study, we set these parameters to *τ* = 24*ms* and *V*_*thr*_ = 1. However, as shown in Fig. 6b, the system decoding performance remains stable across a range of parameter values for the preprocessing LIF. We intentionally chose parameter values that are far from the stability boundaries, even if they are not strictly optimal. Furthermore, working at a higher spiking threshold reduces the number of spikes, which is beneficial for power consumption.

For this study, we evaluated two different frequency band selections: one set of bands, consisting of 60-82 bpm (e1), 82-105 bpm (e2), 105-128 bpm (e3), and 128-150 bpm (e4), was chosen to cover a broad range of heart rate variations, from a relaxed state to tachycardia. In addition, to highlight subtle changes in heart rate and test the system’s ability to handle faster transitions, we also preprocessed the BIDMC dataset using narrower frequency bands: 60-80 bpm (e1), 80-88 bpm (e2), 88-96 bpm (e3), and 96-150 bpm (e4).

### Motion artifact mitigation

Motion artifacts and noise significantly impact PPG signal quality, requiring robust preprocessing for reliable analysis^56^. Common methods include zero-phase Butterworth band-pass filtering (0.5-10 Hz) to remove noise, rolling standard deviation for artifact segmentation, and adaptive thresholding algorithms like a modified Pan-Tompkins for peak detection in challenging environments^45,57^.

In wearable devices, combining PPG with accelerometer data has become standard for addressing motion artifacts^58,59^. Accelerometers detect movement and correlate it with affected PPG segments, enabling precise artifact removal where traditional filters fail. Techniques like adaptive noise cancellation^60^, spectral comparison^61^, and machine learning models further enhance artifact removal by integrating PPG and accelerometer signals^62^.

To explore the dynamics of heart rate estimation in the presence of motion artifacts, we used the Wrist dataset, which includes simultaneous ECG and PPG recordings from eight subjects performing various physical activities. Given the motion-intensive nature of these exercises, the PPG signals were significantly affected by motion artifacts, which posed a challenge for accurate heart rate estimation. Although the dataset does not provide a direct ground truth for heart rate, we utilized the available ECG recordings as a surrogate reference.

**Fig. 7:**
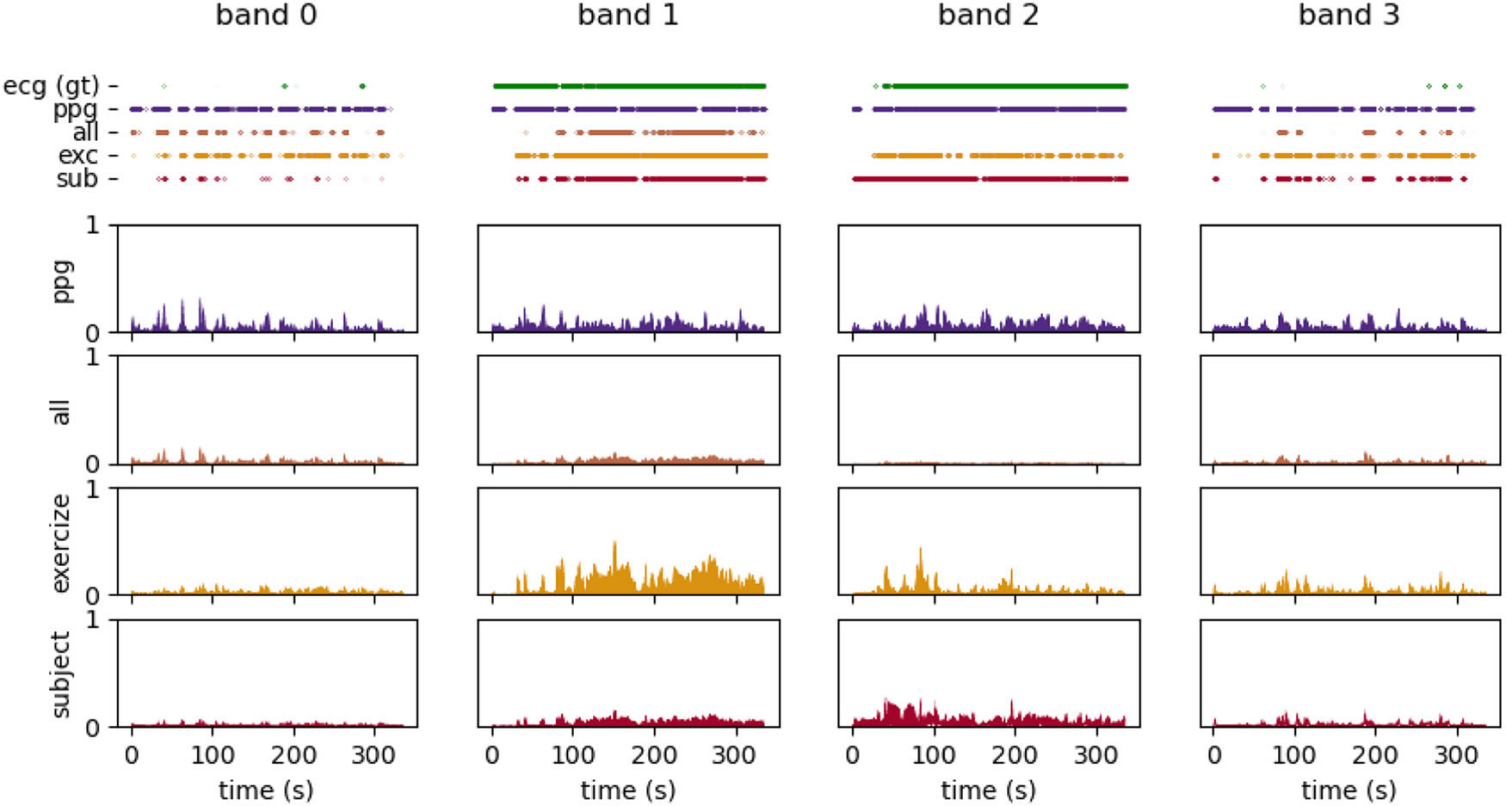
Comparison of cleaned Photoplethysmography (PPG) signals across different processing stages. Each column represents a frequency band. The top row presents the corresponding spiking neuron outputs, illustrating the impact of motion artifact mitigation on neural encoding. Following rows show (from top to bottom) the raw Electrocardiography (ECG) (ground truth), raw PPG, and cleaned PPG signals optimized across all data, subject-specific, and exercise-specific settings.

To mitigate the impact of these motion artifacts, we utilized accelerometer data while addressing the constraints of low-power, always-on systems. In the absence of a true ground truth for heart rate, we leveraged the ECG recordings as a surrogate reference for heart rate estimation. Our methodology optimized a linear combination of the four bandpass-filtered PPG signals and the accelerometer data (one accelerometer per direction). The goal was to align the firing rates of the resulting LIF neuron outputs with those derived from the ECG. A comparison between each band’s ECG (i.e., target), PPG, and cleaned signals—optimized across all inputs, subject-specific, and exercise-specific—is shown in Figure 7, along with the spiking data output from each LIF neuron.

The optimization process employed the Nelder-Mead algorithm^48^, a derivative-free method that is well-suited for optimizing complex, non-differentiable objective functions. The algorithm iteratively refines a set of simplex points to minimize the RRMSE between the firing rate curves derived from the LIF outputs and the reference ECG-derived firing rates. This approach avoids gradient-based methods, which are not ideal due to the overly smooth gradient landscape of the problem. Instead, we used random initialization followed by 20 iterations of optimization to converge on an optimal solution.

This method was applied to optimize the combination of signals either across all data samples or independently per subject or exercise type. Our results indicate that exercise-specific optimization yields performance comparable to subject-specific optimization, which enhances practical applicability. Although this traditional optimization approach is simpler compared to advanced methods, it yielded satisfactory results, balancing computational efficiency with the need for accurate removal of motion artifacts. Other approaches, such as deep learning models, require large amounts of data for training, while offline methods that analyze the full signal dynamics can be computationally expensive and less practical for real-time applications.

### Computational primitives

**E-I balanced networks** provide a stable and adaptive computational basis for all architectures. They consist of tightly coupled excitatory (E) and inhibitory (I) neuronal populations that maintain a dynamic equilibrium between excitation and inhibition. This balance is critical for ensuring stability and preventing runaway excitation or excessive suppression within the network. In such networks, inhibitory neurons provide rapid feedback to counteract excitatory inputs, leading to precise control over the timing and magnitude of neuronal activity. This mechanism supports robust and stable computations while enabling the network to respond adaptively to input fluctuations, making E-I balanced networks fundamental in both biological and neuromorphic systems. Theoretical models^63,64^ suggest that cortical networks operate in a balanced regime, where excitatory and inhibitory inputs dynamically adjust to prevent runaway activity while maintaining responsiveness to stimuli. Mathematically, the total synaptic input to a neuron can be expressed as:

$${I}_{{\rm{total}}}={I}_{{\rm{exc}}}+{I}_{{\rm{inh}}}$$

where *I*_exc_ and *I*_inh_ represent the excitatory and inhibitory synaptic currents, respectively. In balanced networks, these contributions approximately cancel on average, leading to a net input that scales with external drive rather than diverging:

2 $$\langle {I}_{{\rm{exc}}}\rangle \approx \langle {I}_{{\rm{inh}}}\rangle$$

ensuring that fluctuations, rather than absolute magnitudes, dominate neuronal dynamics. The balance is often maintained through inhibitory feedback, which is modeled as:

3 $${I}_{{\rm{inh}}}=g{I}_{{\rm{exc}}}$$

where *g* is the inhibitory gain, typically greater than 1 in cortical networks. This ratio ensures that inhibitory neurons track excitatory activity and stabilize network dynamics.

**Winner-Take-All** is a computational primitive exploiting of competitive neural circuits in which a subset of neurons suppresses the activity of others, allowing only the strongest input to dominate the network’s response^65,66^. These networks are widely used in computational neuroscience and artificial neural systems for decision-making, feature selection, and clustering^41,46,67,68^. The dynamics of a WTA circuit can be described by the firing rate equations:

$$\tau \frac{d{r}_{i}}{dt}=-{r}_{i}+f\left({I}_{i}-\mathop{\sum }\limits_{j\ne i}{w}_{ij}{r}_{j}\right)$$

where *r*_*i*_ represents the firing rate of neuron *i*, *I*_*i*_ is its external input, *w*_*ij*_ are the inhibitory connections between competing neurons, and *f*(⋅) is a nonlinear activation function. The competition arises from strong lateral inhibition, typically modeled as:

$${w}_{ij}=\gamma {{\Theta }}({r}_{j}-{r}_{i})$$

where *γ* controls the inhibition strength and Θ(⋅) is the Heaviside step function, ensuring that only the most active neuron suppresses others. In an idealized case, the network converges to a state where only the neuron with the largest input remains active, while all others are silenced:

$${r}_{k} \,>\, 0,\quad {r}_{j\ne k}=0,\quad \,{\text{where}}\,\quad k={\arg} \mathop{\max }\limits_{i}{I}_{i}.$$

This competitive mechanism enables efficient selection of the most salient input while filtering out weaker signals. Variants of WTA networks incorporate stochasticity, adaptive thresholds, or continuous soft competition to improve robustness and flexibility.

**Neural State Machines** are a class of neural architectures that encode discrete internal states and transition dynamics, enabling robust sequential decision-making, memory, and hierarchical processing^6,47,69^. Unlike traditional feed-forward networks, NSMs maintain internal state representations that evolve over time based on both external inputs and recurrent feedback. The core dynamics of an NSM can be described by a state update equation:

4 $$s(t+1)=f\left({W}_{s}s(t)+{W}_{x}x(t)+b\right)$$

where *s*(*t*) represents the hidden state at time *t*, *x*(*t*) is the external input, *W*_*s*_ and *W*_*x*_ are the recurrent and input weight matrices, respectively, *b* is a bias term, and *f*(⋅) is a nonlinear activation function. State transitions in NSMs often follow a probabilistic or soft-competitive mechanism, where the likelihood of transitioning to a new state depends on an energy function:

5 $$P(s_{t+1} | s_t, x_t) = \frac{\exp(-E(s_{t+1}, s_t, x_t))}{{\sum}_{s^{\prime}} \exp(-E(s^{\prime}, s_t, x_t))}$$

where *E*(*s*_*t*+1_, *s*_*t*_, *x*_*t*_) represents an energy function defining the cost of transitioning between states. This formulation allows NSMs to incorporate probabilistic state transitions, making them robust to noise and uncertainty. To enforce stability and prevent excessive state transitions, an inhibitory competition mechanism is also introduced:

6 $${I}_{{\rm{inh}},i}=g\mathop{\sum }\limits_{j\ne i}{w}_{ij}{s}_{j}$$

where *g* is the inhibitory gain, *w*_*ij*_ represents inhibitory coupling between states, and *s*_*j*_ is the activation of competing states. This ensures that only a subset of states remains active at any given time, preventing unstable oscillations. In addition to their fundamental state-machine-like behavior, NSMs can process asynchronous events directly, without the need for clocked transitions. This capability allows NSMs to take advantage of sparse and slowly changing signals, reducing computational overhead and improving energy efficiency while also taking advantage from the distributed and parallel nature of spiking neural networks, making them more resilient to faults and noise. By combining recurrent dynamics, competitive inhibition, and probabilistic transitions, NSMs provide a powerful framework for modeling adaptive and memory-dependent behaviors in both biological and artificial systems^70^.

### The Neural State Machine architecture

This section introduces the architectures underlying the NSMs, highlighting their unique features and operational principles. Each model builds upon fundamental neuromorphic computing principles to address distinct requirements in encoding and transitioning between heart rate bands (parameters in Table 2).

**Table 2:** Network parameters for the three architectures: Winner-Take-All (WTA), Nearest Neighbors Neural State Machine (nnNSM), and Monotonic Neural State Machine (monoNSM)

	Parameter	WTA	nnNSM	monoNSM
**Neurons per population**	*n*_*exc*0	16	16	16
	*n*_*exc*1	16	16	16
	*n*_*exc*2	16	16	16
	*n*_*exc*3	16	16	16
	*n*_*inh*	16	16	16
	*n*_*exc*_*disinh*	-	16	16
	*n*_*inh*_*disinh*	-	4	4
**WTA connection probabilities**	*p*_*exc*_*exc*_*ex*0	0.85	0.85	0.85
	*p*_*exc*_*inh*_*ex*0	0.40	0.40	0.40
	*p*_*inh*_*exc*_*ex*0	0.50	0.50	0.50
	*p*_*exc*_*exc*_*ex*1	0.85	0.85	0.85
	*p*_*exc*_*inh*_*ex*1	0.40	0.45	0.45
	*p*_*inh*_*exc*_*ex*1	0.45	0.50	0.50
	*p*_*exc*_*exc*_*ex*2	0.85	0.85	0.85
	*p*_*exc*_*inh*_*ex*2	0.40	0.40	0.40
	*p*_*inh*_*exc*_*ex*2	0.50	0.50	0.45
	*p*_*exc*_*exc*_*ex*3	0.85	0.80	0.80
	*p*_*exc*_*inh*_*ex*3	0.40	0.40	0.40
	*p*_*inh*_*exc*_*ex*3	0.50	0.50	0.50
	*p*_*inh*_*inh*	0.20	0.20	0.50
**Input probabilities**	*p*_*inp*_*ex*0	0.30	0.30	0.30
	*p*_*inp*_*ex*1	0.30	0.30	0.30
	*p*_*inp*_*ex*2	0.30	0.30	0.30
	*p*_*inp*_*ex*3	0.30	0.30	0.30
**Disinhibition EI connection probabilities**	*p*_*exc*_*exc*	-	0.50	0.50
	*p*_*exc*_*inh*	-	0.30	0.30
	*p*_*inh*_*exc*	-	0.30	0.30
	*p*_*inh*_*inh*	-	0.50	0.50
**Disinhibition feedback**	*p*_*ex*_*disinh*	-	0.50	1.0
	*p*_*disinh*_*ex*	-	1.0	1.0
	*p*_*ex*_*disinh*0	-	1.0	0.5

This table compares the number of neurons per population and connection probabilities across the different Neural State Machine (NSM) architectures.

This WTA framework is designed to encode distinct heart rate bands using a competitive dynamic among its excitatory populations (e1,e2,e3,e4), as shown in Fig. 3a. Each excitatory population is tasked with representing a specific HR band, and the activation of one population suppresses the activity of others, ensuring only one HR band is active at any given time. The network employs a global inhibitory population (inh) to enforce this competition, where inhibitory signals uniformly suppress all excitatory populations, preventing simultaneous activation. This architecture is suitable for hard transitions between states and is highly effective in competitive environments. However, it lacks the mechanisms necessary for smooth transitions between adjacent states, which can be a limitation when dynamic and gradual changes in HR bands are required.

**Fig. 8:**
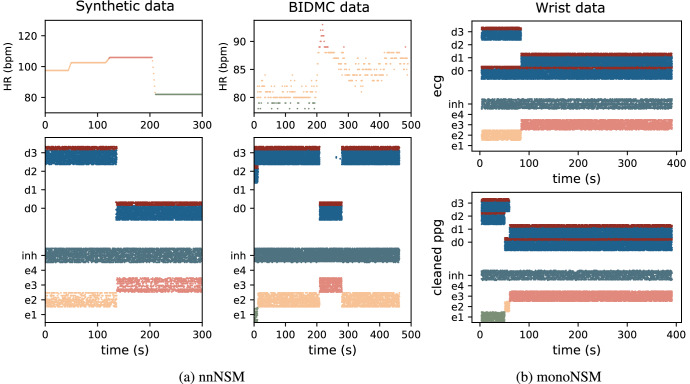
Disinhibition populations activity in Neural State Machines. **a** *Top:* Heart Rate (HR) ground truth for synthetic (left) and real BIDMC (right) dataset. *Bottom:* Rasterplot of the monotonic Neural State Machine network showcasing the disinhibitory populations activity (d0, d1, d2, d3) for both excitatory (blue) and inhibitory (red) neurons together with the excitatory state-populatons (e1,e2,e3,e4) and global inhibition (inh). *Bottom:* activity when presented with cleaned Photoplethysmography (PPG) input. **b** Rasterplot of the monotonic Neural State Machine network showcasing the disinhibitory populations activity (d0, d1, d2, d3) for both excitatory (blue) and inhibitory (red) neurons together with the excitatory state-populatons (e1,e2,e3,e4) and global inhibition (inh). *Top:* activity when presented with Electrocardiography (ECG) input. *Bottom:* activity when presented with cleaned PPG input.

The nnNSM, depicted in Fig. 4a, is based on the WTA architecture augmented with disinhibition mechanisms to enable smooth transitions between adjacent HR bands. The nnNSM architecture consists of excitatory and disinhibition populations. As in the WTA architecture, the excitatory populations are responsible for encoding specific HR bands, with each population dedicated to a distinct frequency range and a global inhibitory population enforces competitive dynamics across the network. Differently to the WTA network, to facilitate smooth transitions and suppress competing activity, each excitatory population is also paired with a disinhibition population (disinh0, disinh1, disinh2, disinh3). The disinhibition populations are composed of an excitatory-inhibitory (E-I) balanced network. This reciprocal inhibition mechanism ensures that the network transitions seamlessly between adjacent HR bands while suppressing activity in non-adjacent bands. Together, the local disinhibition and global inhibition mechanisms enable the network to maintain a balance between sensitivity to input signals and stability in its dynamics. This mechanism avoids abrupt switching and maintains smooth, context-sensitive HR band selection. To further illustrate this, Fig. 8a presents a raster plot of the disinhibition E-I populations and the state network activity in response to biomedical data, highlighting the role of disinhibition in regulating smooth HR band transitions.

The monoNSM, illustrated in Fig. 5, builds upon the WTA architecture, incorporating a hierarchical structure and tailored input routing to support monotonic state transitions. In this setup, the excitatory populations are interconnected through asymmetric lateral inhibitory pathways, enforcing unidirectional transitions between states, which aligns with the monotonic progression. This organization promotes stability and efficient transitions, crucial for dynamic HR tracking under scenarios requiring strict band ordering. The monotonic NSM leverages these mechanisms to offer a robust yet adaptable framework for applications demanding structured state changes while maintaining low computational complexity. Similarly, Fig. 8b displays a raster plot of the state network activity in response to wrist dataset, showcasing how the asymmetric inhibitory pathways enforce ordered state transitions while preserving stability.

### The DYNAP-SE neuromorphic hardware

The neuromorphic processor used in this study is the DYNAP-SE chip, a multi-core asynchronous mixed-signal neuromorphic processor designed to emulate the biophysical behavior of spiking neurons in real-time^15^. Each of its four cores contains 256 Adaptive Exponential Integrate-and-Fire (AdExp-IF) silicon neurons, with each neuron equipped with two excitatory and two inhibitory analog synapses. Synapses in the DYNAP-SE can be configured as slow/fast and inhibitory/excitatory, offering flexible functionality. Additionally, neurons within each core share common bias settings, resulting in shared time constant values. To facilitate communication, each neuron integrates a Content Addressable Memory (CAM) block with 64 addresses, representing its pre-synaptic connections. The chip uses the Address-Event Representation (AER) protocol for asynchronous communication^71^. In this system, each neuron is assigned a unique digital address transmitted asynchronously upon generating an event. The DYNAP-SE’s fully asynchronous inter-core and inter-chip routing architecture supports flexible and precise connectivity with microsecond-level timing, even under heavy system loads.

### Power consumption

The power consumption of the DYNAP-SE cannot be directly measured during online operation, however, we can indirectly assess it as the sum of the required power for spike generation and communication^72,73^, as listed in Table 3^15^:

7 $$P=\mathop{\sum }\limits_{n=1}^{N}{r}_{n}\left({E}_{{\rm{spike}}}\,+\,{E}_{{\rm{enc}}}\,+\,{N}_{{\rm{cores}}}\left({E}_{{\rm{br}}}+{E}_{{\rm{rt}}}\right)\,+\,{N}_{{\rm{cam}}\_{\rm{match}}}\,{E}_{{\rm{pulse}}}\right),$$

where: *E*_spike_, *E*_enc_, *E*_br_, *E*_rt_, *E*_pulse_ are the energy costs estimated via circuit simulations, for generating a spike, encoding and appending destinations, broadcasting within the same core, routing to a different core, and extending the output pulse, respectively. *N* denotes the total number of neurons int he network, *r*_*n*_ is the firing rate of neuron *n*, ${N}_{{\rm{cores}}}$ refers to the number of cores each neuron’s spikes are sent to *N*_cam_match_ is the total number of postsynaptic neurons that receive the input spikes.

**Table 3:** Estimated energy consumption of the DYNAP-SE circuit operations^15^

	Operations	@1.8V
*E*_*spike*_	Generate one spike	883 pJ
*E*_*enc*_	Encode one spike and append destinations	883 pJ
*E*_*br*_	Broadcast events to the same core	6.84 nJ
*E*_*rt*_	Route events to a different core	360 pJ
*E*_*pulse*_	Extend generated pulse	324 pJ

### Performance evaluation metrics

To assess network performance, we employ the RRMSE, a normalized metric that quantifies the deviation between predicted and actual heart rate values. RRMSE is computed as the Root Mean Square-Error (RMSE) divided by the mean of the observed values, providing a scale-independent measure of accuracy. An RRMSE of 0 indicates perfect agreement between predictions and observations. It is defined as follows:

8 $${\text{RRMSE}}\,=\frac{\root\of{\frac{1}{N}\mathop{\sum }\nolimits_{i = 1}^{N}{({y}_{i}-{\hat{y}}_{i})}^{2}}}{\bar{y}}$$

is the mean of the observed values. Specifically, *y* represents the predicted heart rate values, $\hat{y}$ denotes the actual heart rate values (i.r. ground truth), *N* is the total number of observations, and $\bar{y}$ is the mean of the observed values.

### Statistics

All on-chip results were repeated three times on three separate days to ensure robustness. Noise analysis was performed using 10 different seed initializations across the entire synthetic dataset. All reported means were calculated by averaging the mean values across dataset trials for each repeated measurement, while the standard deviations were computed as the average standard deviation across the repeated measurements. To empirically validate the robustness of our approach, we conducted repeated measurements across multiple independent chip initializations on different days. Since each power-on leads to a new parameter configuration, this procedure allowed us to systematically assess the system’s ability to cope with noise and variability in the hardware.

### Network parameters

To ensure reproducibility and facilitate implementation, we provide a comprehensive overview of the key parameters used in our network models. The table below details neuron counts and connection probabilities for three architectures: WTA, nnNSM, and monoNSM. These parameters define population sizes, input connections, and disinhibition mechanisms, serving as a reference for model replication.

## Data Availability

The code to reproduce all experiments and figures in this study is available at https://gitlab.com/neuroinf/monotonic_nsm. Since most of this study has been conducted using the DYNAP-SE board, access to such boards can be made possible upon request from the corresponding authors. All biomedical data used in this study are openly accessible and can be found at the provided citation.

## Code availability

The code to reproduce all experiments and figures in this study is available at https://gitlab.com/neuroinf/monotonic_nsm. Since most of this study has been conducted using the DYNAP-SE board, access to such boards can be made possible upon request from the corresponding authors. All biomedical data used in this study are openly accessible and can be found at the provided citation.
